# Mapping the use of cardiovascular genetic services in pediatric clinical care: challenges and opportunities for improvement

**DOI:** 10.3389/fgene.2024.1476466

**Published:** 2025-01-07

**Authors:** Kerstin Hundal, Courtney L. Scherr, Hoda Fakhari, Sanjana Ramesh, Lisa Dellefave-Castillo, Deb Duquette, Sara Cherny, Elizabeth M. McNally, Gregory Webster, Laura J. Rasmussen-Torvik

**Affiliations:** ^1^ Department of Communication Studies, Northwestern University, Evanston, IL, United States; ^2^ Center for Genetic Medicine, Feinberg School of Medicine, Northwestern University, Chicago, IL, United States; ^3^ Division of Cardiology (Pediatrics), Ann and Robert H. Lurie Children’s Hospital of Chicago, Northwestern University Feinberg School of Medicine, Chicago, IL, United States; ^4^ Department of Preventive Medicine, Feinberg School of Medicine, Northwestern University, Chicago, IL, United States

**Keywords:** clinical genetics, pediatrics, journey mapping, cardiology, genetic integration

## Abstract

**Purpose:**

Clinical genetic testing is increasingly integrated in managing and diagnosing cardiac conditions and disease. It is important to identify ongoing challenges. This study aimed to better understand how genetic testing is integrated into pediatric cardiac care and identify barriers and opportunities for improvement.

**Methods:**

We conducted qualitative interviews with pediatric cardiology clinicians (N = 12). Following a journey mapping approach to data analysis, we described genetic testing workflow phases, participants’ thoughts and behaviors within each phase, and barriers and opportunities for improvement.

**Results:**

Participants described several challenges across the genetic testing workflow, from identifying patients for testing to disclosing results to the patients. Testing logistics, decision-making, and collaboration emerged as the most prominent challenges. Variation remains in the utilization of genetic testing, partially driven by case complexity and type of testing and attributable to other factors, like the level of interaction with genetics experts and inconsistent processes within the electronic medical record.

**Conclusion:**

Clinical genetic pediatric cardiology requires more systematic integration of genetic testing and transparent processes. Major opportunities include the interplay between clinicians, genetic experts, and the EMR. Incorporating process mapping results into clinical logistics may eradicate some barriers experienced by pediatric cardiologists and increase clinical efficiency.

## 1 Introduction

Clinical genetic testing is increasingly integrated into pediatric cardiac care, playing a pivotal role in the management and diagnosis of hereditary aortopathies, dyslipidemias, channelopathies, cardiomyopathies, and congenital heart disease (Musunuru et al., 2020; Landstrom et al., 2021; Helm et al., 2021). Genetic testing results influence diagnostic, prognostic, and therapeutic decision-making, reducing morbidity and mortality (Ahmad et al., 2019; Papadopoulou et al., 2023). For example, positive genetic testing results for familial hypercholesteremia can confirm a diagnosis and impact therapeutic choices (Musunuru et al., 2020). In addition, genetic testing results can aid in identifying at-risk family members through cascade testing. Genetic information can guide risk mitigation strategies, including medication use and surgical intervention, and influence lifestyle choices (Ahmad et al., 2019).

Despite these benefits, genetic testing in pediatric cardiac care remains challenging. The diagnostic and predictive yield of genetic testing differ greatly by condition, and not all genetic bases of disease are well-understood (Ingles et al., 2020). Genetic information may or may not provide clues to the probability of a potential disease diagnosis, the age at which the disease might manifest, and the clinical utility of test results (Landstrom et al., 2021; Stafford et al., 2022). Pediatric clinicians must carefully weigh the risks and benefits and consider the broader ethical and psychosocial consequences of genetic testing (Botkin et al., 2015; Greene et al., 2024).

Genetic testing involves coordinating logistics (*e.g.*, when and what tests to order), educating and consenting patients, interpreting genetic information, and communicating test results to patients and families (Landstrom et al., 2021; Greene et al., 2024; Berrios et al., 2021). Although most clinicians view genetic testing positively, many encounter difficulties integrating it across the care continuum (Scherr et al., 2022; Lopez Santibanez Jacome et al., 2022; Vadaparampil et al., 2015). Previous studies have investigated genetic testing processes in adult health contexts and with other clinicians, especially oncologists and primary care clinicians (Lopez Santibanez Jacome et al., 2022; Donohue et al., 2021; Mazzola et al., 2019; Hauser et al., 2018; Scherr et al., 2020). However, a knowledge gap exists in pediatric cardiology, where the logistics and ethics are more complex.

We used journey mapping as a qualitative analytical tool to investigate how pediatric cardiology clinicians currently use genetic testing in patient care. Given the focus on clinicians and processes, we use the general term “patients” in this manuscript to refer to patients and caregivers. This study aims to answer the following research questions:

RQ1: How is genetics currently used by clinicians working in pediatric cardiology settings?
RQ2: What are pediatric cardiology clinicians’ experiences using genetics in their practice?
RQ3: What are current challenges and opportunities for improvement along the care trajectory?

## 2 Methods

### 2.1 Participants and recruitment

The present inquiry is part of a broader study that conducted qualitative interviews with cardiovascular clinicians to inform the development of an educational program about genetics in Sudden Cardiac Death (SCD) (Scherr et al., 2022). Following IRB approval (STU00210365), participants were recruited from cardiology practices in the Midwest and the Northeast, using purposive sampling. An expert group of health clinicians, researchers, and genetic counselors identified potential participants and sent recruitment emails. Interested participants were eligible if they were (1) employed by an accredited hospital or health system in the United States, (2) affiliated with cardiology, (3) involved in the care of patients at risk for SCD, (4) a physician (MD) or advanced practice nurse (APN), and (5) able to read and speak English. Eligible participants were invited to complete a consent form, a short demographic survey, and an individual online interview.

Given the focus of this study on pediatric cardiac care, we only included interviews conducted with pediatric MDs or APNs working at one children’s hospital in the Midwest (*n* = 12). We focused on one hospital due to variability in cardiac genetic practices. The children’s hospital is a teaching hospital in an urban setting. Participants in this study have access to a cardiovascular genetics clinic located within the hospital and a board-certified genetic counselor with pediatric cardiac genetics expertise.

### 2.2 Data collection

Two researchers trained in qualitative research methods (KH and HF) conducted the interviews between December 2019 and November 2020. The interviews lasted approximately 30-minutes and were audio recorded, professionally transcribed, de-identified, and checked for accuracy. In appreciation for their time, participants could choose between a $25 gift card or a $25 donation to the Sudden Arrhythmia Death Syndromes Foundation.

### 2.3 Data analysis

The present study is a secondary analysis of the interview data. We limited our analysis to questions about clinicians’ use of genetic testing and descriptions of barriers and facilitators. Our analysis followed a journey mapping approach based on user-centered design principles that is increasingly utilized in health services research (Madathil et al., 2020; Joseph et al., 2020). Journey mapping serves as a strategic tool for understanding patients’ or clinicians’ experiences within the healthcare system. It reveals challenges and points for improvement via a visual representation with a horizontal axis depicting chronological events and a vertical axis capturing actions by phase. It typically requires a comparatively low sample size—most studies following this approach include less than ten participants (Madathil et al., 2020).

MaxQDA version 20 facilitated data analysis. (VERBI Software, 2021) The first step involved mapping out clinicians’ genetics workflow on the horizontal axis. To achieve this, two researchers read through all transcripts and used open coding to identify distinct phases of the genetic testing workflow. The resulting codes informed the development of an initial codebook. The two researchers then applied the codes to three transcripts, discussed discrepancies, and revised the codebook. The final codebook was applied to five additional transcripts. After achieving acceptable intercoder reliability (α = 83.0) (Krippendorff, 2006), the remainder of the transcripts were divided between coders. The workflow’s resulting phases were mapped horizontally to represent a chronological sequence of events.

In the second step, thematic analysis (Braun and Clarke, 2006; Vaismoradi et al., 2013) was guided by the overarching research questions, which focused on clinicians’ experiences, challenges, and potential improvement points. The two researchers read through the data pertaining to each of the phases identified in the previous step and applied open coding. The resulting codes were discussed and revised following a consensus-based approach and then consolidated to represent more prominent themes. Coding disagreements were resolved through consultation of the second author (CLS). The themes were mapped vertically to represent clinicians’ experiences across the genetic testing workflow.

## 3 Results

### 3.1 Participant characteristics

This was a cohort (N = 12) of young practitioners (mean age of 38.1), including seven residents or fellows in training (58.3%), two attendings (16.6%), and three advanced nurse practitioners (APNs, 25%). Approximately half were female (*n* = 7; 58.3%), the majority were white (*n* = 11; 91.6%), and all were non-Hispanic. Participants reported working less than 5 years at their current practice (*n* = 9; 75%) and had less than 5 years of training in cardiology (*n* = 7; 58.3%). Participants spent most of their time in full-time patient care (*n* = 6; 50%) (see Table 1).

**TABLE 1:** Sample characteristics.

Variable	*n*	%
Gender		
Female	7	58.3
Male	5	41.7
Age (*M, SD*)	38.08 (9.79)	
Race/ethnicity		
Asian Non-Hispanic	1	8.3
White Non-Hispanic	11	91.7
Training		
MD		
Resident	1	8.3
Fellow	6	50.0
Attending	2	16.6
APN	3	25.0
Years of training as healthcare professional		
Less than 5 years	0	0
5–10 years	9	75.0
11–15 years	1	8.3
16–20 years	0	0
More than 20 years	2	16.7
Years of training in cardiology		
Less than 5 years	7	58.3
5–10 years	1	8.3
11–15 years	1	8.3
16–20 years	2	16.7
More than 20 years	1	8.3
Years of work at current practice		
Less than 5 years	9	75.0
5–10 years	1	16.7
11–15 years	0	0
16–20 years	0	0
More than 20 years	1	8.3
Percentage of time spent with patient care		
1%–25%	1	8.3
26%–50%	1	8.3
51%–75%	4	33.3
76%–100%	6	50.0

### 3.2 Qualitative results

**FIGURE 1:**
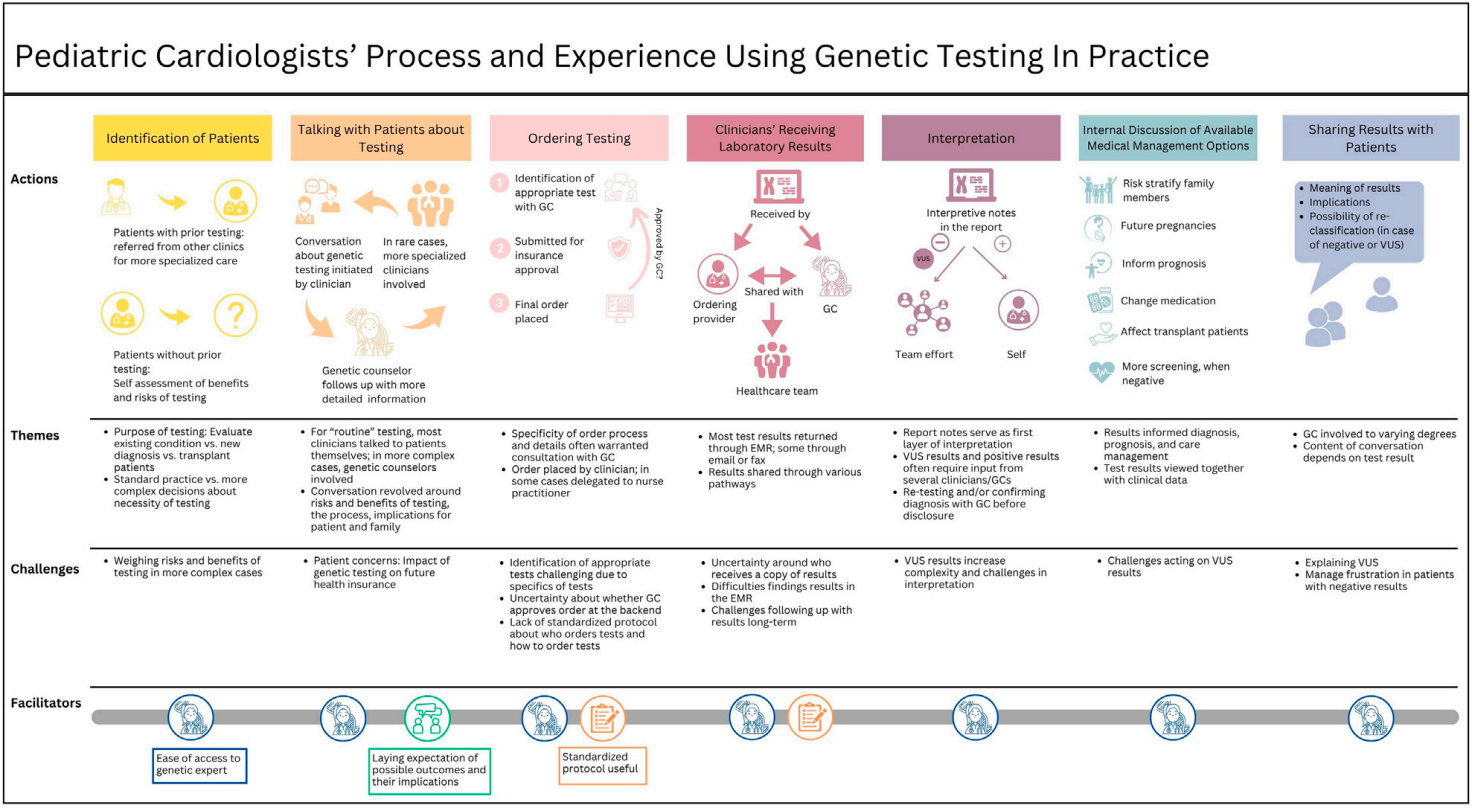
Journey map outlining pediatric clinicians’ workflow and experiences using genetic testing in practice.

We identified seven workflow phases: (1) identifying patients for genetic testing, (2) talking with patients about getting genetic testing, (3) ordering genetic testing, (4) clinicians receiving laboratory results, (5) interpreting test results, (6) discussing medical management options within the healthcare team, and (7) disclosing test results to the patient. Each phase is discussed separately below and includes descriptions of participants’ experiences, challenges, and points for improvement. For the complete journey map, see Figure 1. Participant’ training characteristics are noted after quotes, with MD for physicians and APN for advanced practice nurses, followed by the training position (i.e., resident or fellow).

#### 3.2.1 Identifying patients for genetic testing

The first phase in the genetic testing workflow involves identifying patients who may benefit from genetic testing. Some participants managed patients who had undergone genetic testing elsewhere and were referred to them for more specialized care. In most other cases, participants reported determining the appropriateness of genetic testing for their patients. The extent to which genetic testing was integrated into clinical practice varied based on cardiac condition and patient characteristics. For some conditions, such as cardiomyopathies or aortopathies, ordering genetic testing was considered standard practice: “When I see patients with cardiomyopathy or heart muscle disease problems, we almost always send a genetic panel to try to uncover the cause of the disease” (MD, Fellow). A genetic counselor was often involved in cases that required more complex evaluations.

Participants described two primary reasons for recommending genetic testing. It was commonly recommended for patients with a clinical diagnosis of a heritable cardiovascular disease, such as cardiomyopathies or arrhythmic disorders, to determine the cause and guide management. In other instances, genetic testing was ordered to evaluate patients for a new cardiac disease or to confirm a suspected diagnosis, as described by one participant:

Sometimes it's because they have a concern for a new diagnosis of a cardiomyopathy. They’ve been out in the world living their life not knowing anything’s wrong, then they come into us because they’ve become symptomatic from a new heart problem. Then lo and behold, they have some degree of heart failure, so they’re getting worked up for a handful of different possibilities, and an underlying genetic problem is in the mix. (MD, Fellow).

This type of diagnostic testing was commonly done on children who were admitted to the CICU for sudden collapse or cardiac arrhythmia, or for children with aortopathies. Some participants reported ordering chromosome microarrays (CMA) to screen newborns with congenital heart defects for copy number variants (*i.e.*, missing or extra chromosome material).

#### 3.2.2 Talking with patients about getting genetic testing

Once patients were identified as potentially benefiting from genetic testing, participants discussed the option with them. The conversation was described as a multi-step process involving different types of clinicians at various time points, depending on the case’s complexity. For microarrays for newborns, many clinicians talked directly with the patients without involving other clinicians:

So if we’re going to send just basic genetic testing, like a microarray, then I would talk to the family about that. If we’re actually looking for very specific gene markers, I want to do anything more extensive than that, then I would refer to [genetic counselors]. (APN).

For patients who needed more complex genetic testing such as gene panels, exome, or genome, the clinician offered basic information and counseling, followed by a more in-depth discussion, generally led by a genetic counselor, and sometimes combined with or followed by another discussion with a specialized clinician. “The nitty-gritty is usually done by either the subspecialty service who is evaluating the patients, so whether it be the electrophysiology team, or the cardiomyopathy team, or by the geneticist if they’re in the mix already” (MD, Fellow). Within those conversations, participants acknowledged that genetic testing results may have implications for family members. Some also addressed financial concerns: “Some families are a little hesitant because if something comes up, it could affect that person’s insurance in the future even if they don't have the disease” (MD, Fellow).

One participant highlighted the importance of preparing patients for the implications of their test results and medical management decisions in the initial discussion to avoid confusion and anxiety post-testing: “I mean usually I’ve at least given them a reason as to why I am doing the test before we actually get it done. To sort of lay the groundwork for the potential outcomes that it could be” (MD, Attending).

#### 3.2.3 Ordering genetic testing

After consent, most participants involved a genetic counselor or geneticist if they were not already involved: “[The genetic experts] say for us to get it ordered just right, there is a particular way it needs to be done, and sometimes I’ll include them and either have them help me order it or have them order it.” (MD, Fellow). One challenge was the identification of appropriate tests. Typically, participants consulted a genetics expert who told them what test to order. Regardless of who identified the appropriate test, most participants believed that a genetics expert approved the order. However, there was some uncertainty about whether this was true for all tests.

Once the appropriate test was identified, the order for testing was placed and insurance approval was obtained, although this may differ between inpatient and outpatient testing. The person ordering was commonly chosen on a case-by-case basis, which made one participant wish for a more standardized protocol: “We would love to have one, but there’s no standardized protocol. We don't have the staff for that” (APN). After the order was submitted, test results were typically returned within several days (for rapid inpatient testing) or several weeks (for outpatient testing).

#### 3.2.4 Clinicians receiving laboratory results

Most test results were returned to the clinician through the electronic medical record (EMR) system, although one participant noted that in rare cases, they were returned via email or fax. Participants described various pathways in which they received a copy of the test results and how they were shared with the healthcare team. In many cases, a genetic counselor first received the results and then informed the healthcare team: “The genetic counselor usually knows about the results first and will inform me about the result and its significance. But also I can see this through their Epic chart” (MD, Fellow). In other cases, the ordering clinician received a notification through the EMR and then discussed the results with the genetics team. Sharing and discussing results commonly occurred through spontaneous face-to-face encounters on the floor: “We are always bumping into one another so … it's really easy to find them. If you send it to them in your EMR they may or may not see it right away, but if you need some information from them faster than that you just have to knock on their door.” (MD, Fellow).

Several participants expressed uncertainty about who received a copy of the results: “The ones I’ve ordered, if it's an outpatient, I typically have gotten back into my in-basket. Now whether or not the genetics folks get a separate copy or not when it comes back, I’m not certain” (MD, 2). The same participant described difficulties finding results.

The results are updated in our media tab, so we would just have to search through it. Sometimes it is a little bit difficult to find. Once we find it out, we would put it in our most recent notes, so that it gets forwarded. (MD, Attending).

Two participants reported challenges with test results that took several weeks to obtain, although this occurred rarely.

#### 3.2.5 Interpreting test results

Results interpretation was described as a team effort. The report provided detailed information commonly used for initial interpretation. When reports were not complete enough, participants would contact the testing company to request more information. Most participants then consulted the genetics team, and some a genetic expert to help interpret the report or confirm their interpretation. The extent to which participants consulted other specialized clinicians depended on the complexity of the results and participants’ familiarity with the test. One participant explained.

If they fall into one of the handful of genetic mutations that we see all the time and we treat all the time, take care of all the time, we’re pretty comfortable with it, but I would probably get out of my comfort zone pretty quickly if it was just all newcomers for genetic mutations. (MD, Fellow).

Others needed help understanding the results when they involved a variant of uncertain significance (VUS) which cannot be used for diagnosis or medical management decisions due to lack of information about the potential pathogenicity of the variant.

In those cases, I really do rely on the genetic counselor because there are some VUSs that are felt to be a little bit more suspicious than others, in which case they might recommend testing family members to try to get more data. (MD, Fellow).

Participants often managed negative test results without the help of a genetic expert. However, the ease of access to a genetics expert was described as invaluable to the process of interpreting genetic testing results.

#### 3.2.6 Discussing medical management options within the healthcare team

Commonly, positive test results had less of an impact on the patient’s medical management than on their family members. “Our patients are mostly pediatrics, so it doesn't directly help the patients immediately, but it helps us risk stratify or evaluate the other family members to see if they also have the potential to develop a heart disease” (MD, Resident).

Sometimes, positive test results directly impacted the patient and were used to inform prognoses, adjust medication, or even alter newborn nutrition. For example, one participant explained: Occasionally the genetic testing can give us some idea as to what to expect with the disease because certain mutations in certain genes have better or worse prognosis (MD, Fellow). Even if there is a negative genetic test result, participants noted the value of clinical cardiac screening for family members. For example, one participant explained:

So if a patient has the disease but has a negative genetic test, then unfortunately, that tells me we just don't know what caused their disease. […] And so in those cases we have the family members continue to get periodic screening with Echo [and] EKG, since we can't rule in or rule out their risk for disease using genetics. (MD, Fellow)

All participants reported consulting a genetic expert to help interpret and make sense of VUS results. Additional research and considerations of clinical factors were necessary for optimal decision-making. One participant explained:

If there’s some inkling that it [VUS] may be an actually pathogenic variant we can’t confirm it, but that’s a suspicion, then we’re probably more likely to consider it to be pathogenic given the clinical context. Then when it's truly a VUS, like we have no idea, there’s nothing that we know about it, it is noise but it doesn't actually, probably contribute much to what we’re doing. (MD, Fellow).

#### 3.2.7 Disclosing test results to the patient

The last step of the workflow involved disclosing test results to the patients and their families. Only one participant reported communicating test results to the family independently; all others relied on a genetic counselor to support or conduct the conversation. One participant described approaching the genetic counselor before talking with the family.

If it's positive, I usually talk to genetic counselor first and figure out if there’s anything specific about the counseling that I would need to know in terms of how she would counsel them, and if in talking with her I find out that this discussion is going to be more complex or is not going to be a straight forward, then I will usually have her do it or have her do it with me. (MD, Fellow).

Participants appreciated the presence of genetic counselors and their knowledge and counseling skills.

I say our genetic counselors, they do a lot of research before they come to talk to us and the family so that they have a lot of background on whatever it is that comes up. They always come with a lot of information because we forget these things in between patients, to be perfectly honest. (APN).

The discussion was informed by the type of result. The return of positive results typically included an explanation of the variant, the effect of test results on medical management and prognosis, and a discussion of implications like future pregnancies and cascade testing for at-risk family members. For negative test results, participants explained that there was no identifiable genetic cause of the disease or condition at this time and that they would continue their periodic medical checkups. Several participants described the field of genetics as rapidly evolving, which may change the implications of a negative test result:

I usually just say that obviously the field in and of itself is exploding and our knowledge is growing. There would be new diagnoses and things and if new information becomes available, I’ll obviously bring that to their attention. (MD, Attending).

The disclosure of test results was more complex when VUS were involved. All but one participant reported deferring to a genetic counselor when VUS results were involved because of the inherent uncertainty in these results and the belief that VUS require more explanation. Discussions typically included an explanation of VUS and its potential for re-classification:

I'll tell them that we found variants that aren't necessarily at this time associated with what were their underlying heart disease in terms of what we were looking for … but if it's variants that seem potentially significant, then I mean we definitely involve genetic counseling to explain more about what that could mean for this patient. (MD, Fellow)

## 4 Discussion

The analysis of participants’ experiences using genetic testing in pediatric patient care generated a visual map describing the different phases of the genetic testing workflow (Figure 1). The mapping process revealed that participants value and frequently use genetic testing in patient care but also face challenges.

Consistent with prior studies, most participants viewed genetic testing as an important aspect of cardiac patient care that could inform diagnosis, prognosis, and care management (Ahmad et al., 2019; Papadopoulou et al., 2023; Scherr et al., 2022). A prior study found variations in the extent to which genetic testing was integrated into pediatric cardiac care across several sites in the United States, with the testing frequency ranging from none to 97% of patients (Ware et al., 2021). All participants in the current study reported ordering genetics in the care of their patients. Given genetic counselors’ central role in the workflow, more consistent integration may be attributed to the presence of a genetics clinic within the cardiology department, providing easy access to genetic experts.

Several challenges persist, such as a lack of standardization. Decision-making about genetic testing and the involvement of genetic specialists often was at the discretion of individual clinicians, leading to variability in genetic testing and counseling. While genetic testing was considered routine for certain conditions like cardiomyopathies, its application in other cases required careful consideration. Participants differed in the extent to which they consulted genetic experts. Some participants collaborated closely with the cardiovascular genetics team throughout the genetic testing workflow, while others sought input as needed. There is a longstanding discussion in the genetics community about the benefits and limitations of genetic testing ordered by non-genetics providers, including in the cardiovascular genetics setting (Feldman et al., 2023; Muller et al., 2020; Helm et al., 2018). This is a complex discourse which is impacted by a number of factors, including access to genetic counselors and financial support for their positions in healthcare institutions, the very broad range genetics expertise in non-genetics providers (from very little to true expert), and the rapidly evolving pace of genetic medicine (Arscott et al., 2016; Hoskovec et al., 2018). Overall, we found that participants reported a positive view of genetic counselors in their practice, and commonly relied on them, especially when interpreting VUS results. Reports of VUS are common, and as the breadth of genetic testing options increases, more VUS are likely (Cherny et al., 2021). The frequency and complexity of explaining them to patients highlight the importance of sufficient patient education and consent during pre-test counseling. As our participants indicated, providing patients with such information can streamline result disclosure and enhance patient comprehension regarding the implications of those results (Findley et al., 2023).

A recent study documented the varied practice models across and within pediatric cardiology clinics (Rickman et al., 2023). We found significant variation in a single hospital’s workflow, partly driven by each case’s complexity. However, we found variations attributable to other factors, like inconsistencies in working with genetics experts and processes within the EMR. Many participants relied on *ad hoc* methods, such as informal encounters on the floor or during rounds, to follow up on orders or share results with colleagues and genetic experts. Notably, these consultations were largely possible due to the proximity of the cardiovascular genetics team to the pediatric cardiology clinic. Despite the accessibility of genetics experts, following up with results long-term remained challenging. Managing genetic testing was further complicated by EMR system limitations, making finding the results time-consuming. Participants were unsure about how the data was managed and shared with the larger healthcare team.

Previous studies found that streamlining the genetic testing workflow and integrating genetic testing results into the EMR can increase clinical efficiency and improve patient care (Rasmussen et al., 2019; Mohananey et al., 2023). Our findings also emphasize the need for a more systematic integration of genetic testing and a more transparent process that outlines the interplay between clinicians, genetic experts, and the EMR in pediatric cardiac care. Being clear about the roles of different clinicians and the EMR in managing genetic testing may eradicate some of the barriers experienced by pediatric cardiologists and increase clinical efficiency. Furthermore, artificial intelligence holds promise in streamlining the genetic testing workflow and assisting with test result classification, although its clinical adoption in routine care has yet to be investigated (Kearney et al., 2020).

### 4.1 Limitations

Most of our participants were in training, had worked less than 5 years at their current practice (*n* = 9; 75%), and had access to a dedicated cardiovascular genetics team. While this map may not be valid for more experienced clinicians or those working outside a tertiary care center, it represents a cohort of young clinicians who are the future of clinical medicine with easy access to genetic experts. Importantly, all data were collected from one children’s hospital in the Midwest that has access to a cardiovascular genetic clinic and a board-certified genetic counselor. The study findings may differ in clinics with varying levels of access to genetic counselors and related resources. Furthermore, this work provides novel data specific to pediatric cardiology, but future studies will be required to compare and extrapolate these data to other healthcare systems and disciplines. Finally, this was a secondary analysis of data; the interview guide was not optimized for an in-depth exploration of thoughts and emotions, which is typical of the journey mapping methodology.

### 4.2 Conclusion

Developing standardized protocols, streamlining the genetic testing and genetic counseling workflow, and enhancing EMR capabilities may improve clinical efficiency, increase genetic testing uptake, and ultimately enhance patient care.

## Data Availability

The raw data supporting the conclusions of this article will be made available by the authors, without undue reservation.
